# True Durability: HIV Virologic Suppression in an Urban Clinic and Implications for Timing of Intensive Adherence Efforts and Viral Load Monitoring

**DOI:** 10.1007/s10461-014-0917-6

**Published:** 2014-11-05

**Authors:** Debra A. Benator, Angelo Elmi, Manuel D. Rodriguez, Howard B. Gale, Virginia L. Kan, Heather J. Hoffman, Susan Tramazzo, Karen Hall, Angela McKnight, Leah Squires

**Affiliations:** 1 Infectious Diseases Section, Medical Service, Veterans Affairs Medical Center, 50 Irving Street NW, VAMC, 151B, Washington, DC 20422 USA; 2 Division of Infectious Diseases, The George Washington University, Washington, DC USA; 3 Milken Institute School of Public Health, The George Washington University, Washington, DC USA; 4 Psychology Service, Veterans Affairs Medical Center, Washington, DC USA

**Keywords:** HIV, Virologic suppression, Viral rebound, Durability of virologic suppression, Adherence

## Abstract

Although the majority of HIV-infected patients who begin potent antiretroviral therapy should expect long-term virologic suppression, the realities in practice are less certain. Durability of viral suppression was examined to define the best timing of targeted adherence strategies and intensive viral load monitoring in an urban clinic population with multiple challenges to ART adherence. We examined the risk of viral rebound for patients who achieved two consecutive viral loads lower than the lower limit of quantification (LLOQ) within 390 days. For 791 patients with two viral loads below the LLOQ, viral rebound >LLOQ from the first viral load was 36.9 % (95 % CI 32.2–41.6) in the first year, 26.9 % (95 % CI 21.7–32.1) in the year following one year of viral suppression, and 24.6 % (95 % CI 18.4–30.9) in the year following 2 years of viral suppression. However, for patients with CD4 ≥300 cells/µl who had 3–6 years of virologic suppression, the risk of viral rebound was very low. At the population level, the risk of viral rebound in a complex urban clinic population is surprisingly high even out to 3 years. Intensified monitoring and adherence efforts should target this high risk period. Thereafter, confidence in truly durable virologic suppression is improved.

## Introduction

HIV-1 RNA viral load (VL) monitoring is currently recommended every 3–4 months for patients on antiretroviral therapy (ART). Among those patients with suppressed viral load for greater than 2 years, monitoring at 6 months intervals is considered reasonable [1]. As these guidelines are based predominantly on clinical trials and on expert opinion, our objective was to examine the risk of viral rebound over time in a large urban HIV Clinic, and better define the durability of virologic suppression and its implications for viral load monitoring and for targeted adherence strategies.

## Methods

The objective of the analysis was to describe the risk of rebound among patients with virologic suppression. We used the HIV Clinical Case Registry to describe the population of patients with HIV infection who had at least one outpatient visit to the Washington DC Veterans Affairs Medical Center from January 1, 2005 to December 31, 2011. We evaluated every paired HIV-1 viral load (VL) and CD4 count performed by the Infectious Diseases Laboratory during the period of observation. Time to rebound was computed using consecutive sequences of observations for subjects whose initial two viral loads were below the lower limit of quantification (LLOQ) and had a viral load measurement within 390 days. Although the median frequency of viral load monitoring for the clinic between 1999 and 2011 was 113 days (IQR; 96–138), we aimed to be inclusive of those patients with more minimal monitoring up to a maximum of approximately 13 months between measurements.

Two analyses were performed. In Analysis A, viral rebound was defined as a viral load greater than the LLOQ. In Analysis B, viral rebound was defined as a viral load greater than 200 copies/ml. Subjects were classified as censored either if they reached the end of the study while remaining virally suppressed, or if at some point a gap of 390 days between tests occurred. Only the first period of virologic suppression for each patient was included in these analyses.

Kaplan–Meier and life table curves were generated to describe time to viral rebound in the cohort. Analyses were done on the cohort as a whole and also as stratified by CD4 groups <300 cells/µl and >300 cells/µl at the time of inclusion. Homogeneity of survival curves in the latter case was tested via the log rank test. Cox’s Proportional Hazards model was also used to quantify the degree and direction of relative risk between CD4 groups. The proportional hazards assumption was assessed visually using log–log survival plots. Analysis was performed using the lifetest, phreg, and freq procedures (SAS 9.3, Cary, NC), and data management was performed using R 3.0.1.

## Results

From January 2005 to December 2011, 1544 patients had at least one outpatient visit. Among these patients, 97 % were male, 75 % were black or African American, and the median age was 50 years. Reported risks for exposure to HIV included sex with a male (30 %), sex with a female (50 %) and injection drug use (20 %). Approximately 30 % of patients were co-infected with Hepatitis C and other co-morbid illness was common including drug and alcohol dependence and mental health disorders in approximately 50 % of patients. Approximately 75 % of patients received antiretroviral therapy (ART) during this period. Among those on ART, 30 % received a non-nucleoside reverse transcriptase inhibitor (NNRTI) based regimen (95 % efavirenz-based), 30 % received a protease inhibitor based regimen (85 % boosted with ritonavir and 15 % unboosted), and 3 % received an integrase inhibitor or other regimen. The remaining 37 % of patients either switched drug classes during this period or received a regimen consisting of three or more drug classes, predominantly nucleosides, NNRTIs and boosted PIs.

During 2005–2011, there were a total of 14,434 paired VL and CD4 results from 1355 patients. Of these, 791 subjects met the criteria of two consecutive viral loads below the LLOQ within 390 days, then followed by at least one subsequent viral load.

### Overall Risk of Viral Rebound

#### Analysis A: Viral Rebound Defined as >LLOQ

Among our cohort of 791 patients, 431 (55 %) experienced viral rebound. Of the 360 patients with sustained viral suppression, 317 (40 % of our cohort) were censored having reached the end of the study period without experiencing rebound, and 43 (5 % of the cohort) were censored due to having had a gap of at least 390 days between testing.

#### Analysis B: Viral Rebound Defined as >200 Viral Copies/ml

334 patients experienced rebound with detectable viremia >200 copies/ml. Of the remaining 457 patients with viral suppression, 404 were censored having reached the end of the study period without rebound and 53 for having had a testing gap of 390 day or more (Table 1).

**Table 1:** Summary of censoring and events by CD4 strata and rebound definition

Analysis A, rebound defined >LLOQ	*n*	Sustained viral suppression	Viral rebound >LLOQ	Censored by testing gap >390 days
<300 cells/μl	248	73 (29 %)	163 (66 %)	12 (5 %)
>300 cells/μl	543	244 (45 %)	268 (49 %)	31 (6 %)
Total	791	317 (40 %)	431 (55 %)	43 (5 %)

Analysis B, rebound Defined > 200	*n*	Sustained viral suppression	Viral Rebound >200	Censored by testing gap >390 days
<300 cells/μl	248	101 (41 %)	133 (54 %)	14 (6 %)
>300 cells/μl	543	303 (56 %)	201 (37 %)	39 (7 %)
Total	791	404 (51 %)	334 (42 %)	53 (7 %)

*LLOQ* lower limit of quantification

### Risk of Rebound by CD4 Strata

**Fig. 1:**
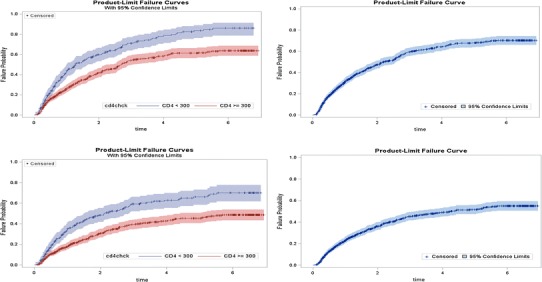
*Top panels* Probability of viral rebound above lower limit of quantification (LLOQ) where the *right panels* is the cohort as a whole and the *left panels* is stratified by CD4 groups. *Bottom panels* Probability of viral rebound above 200 where the *right panels* is the cohort as a whole and the *left panels* is stratified by CD4 groups

As shown in Fig. 1, in an unadjusted Cox Proportional Hazards model, the hazard ratio for rebound comparing patients with CD4 ≥300 cells/μl to those with CD4 < 300 cells/μl was 0.571 (95 % CI 0.470–0.693) for Analysis A, >LLOQ and 0.54 (95 % CI 0.44, 0.68) for Analysis B, >200. The log-rank statistic which formally tests whether the Survival Curves for each group overlap was highly significant (χ^2^ = 32.26 for >LLOQ and 29.99 for >200, *p* < 0.0001).

### Risk of Viral Rebound by Year for Analysis A, >LLOQ

**Fig. 2:**
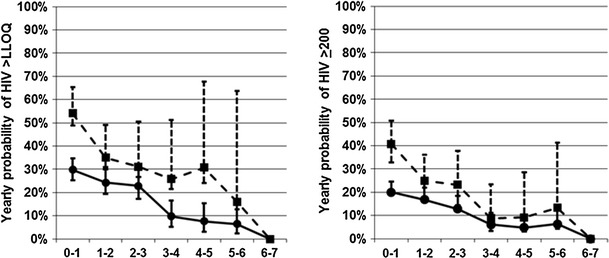
Yearly probability of viral rebound (*left*) HIV >lower limit of quantification (LLOQ) and (*right*) HIV >200. *Dashed line square* CD4 <300, *Solid line circle* CD4 ≥300

As shown in Table 2 and Fig. 2, the risk of early virologic failure >LLOQ was high. For patients with two viral loads below the LLOQ, virologic rebound from the first viral load was 36.9 % (95 % CI 32.2–41.6) in the first year, 26.9 % (95 % CI 21.7–32.1) in the year following one year of viral suppression, and 24.6 % (95 % CI 18.4–30.9) in the year following two years of viral suppression. When stratified by CD4 cell count, patients with >300 cells/μl were at lower risk of viral rebound than those with <300 cells/μl: 29.8 % (95 % CI 28.3–35.2) versus 54.1 % (95 % CI 48.8–65.4) in the first year, 24.2 % (95 % CI 22.9–30.7) versus 35.0 % (95 % CI 31.0–49.2) in the second year and 22.9 % (95 % CI 21.3–30.8) versus 31.1 % (95 % CI 26.7–50.4) in the third year. This difference achieved statistical significance in the first year and in the second year at *p* < 0.05.

**Table 2:** Probability of HIV rebound above the lower limit of quantitation during continuous suppression

Initial CD4 count	Years of HIV suppression (year)	No. at start of Year	No. of rebounds	Yearly %Probability (95 % CI)	Cumulative % Probability (95 % CI)
<300 cells/μl	0–1	248	99	54.1 (48.8–65.4)	43.6 (37.2–50.1)
	1–2	118	32	35.0 (31.0–49.2)	59.9 (53.1–66.4)
	2–3	65	16	31.1 (26.7–50.4)	70.9 (63.8–77.2)
	3–4	38	8	25.8 (21.6–51.3)	77.8 (70.5–83.7)
	4–5	24	6	30.8 (24.1–67.8)	83.5 (76.2–88.9)
	5–6	15	2	16.0 (12.8–63.7)	86.0 (78.5–91.1)
	6–7	10	0	0.0	86.0 (78.5–91.1)
>300 cells/μl	0–1	543	131	29.8 (28.3–35.2)	25.9 (22.2–29.8)
	1–2	337	68	24.2 (22.9–30.7)	41.9 (37.4–46.5)
	2–3	224	43	22.9 (21.3–30.8)	54.1 (49.2–58.9)
	3–4	152	13	9.7 (9.2–16.8)	58.4 (53.4–63.2)
	4–5	115	8	7.7 (7.3–15.3)	61.3 (56.2–66.2)
	5–6	94	5	6.5 (6.1–15.5)	63.7 (58.5–6.86)
	6–7	61	0	0.0	63.7 (58.5–68.6)
All combined	0–1	791	230	36.9 (32.5–41.9)	31.4 (28.1–34.8)
	1–2	455	100	26.9 (22.1–32.6)	47.5 (43.7–51.3)
	2–3	289	59	24.6 (19.1–31.7)	59.3 (55.2–63.2)
	3–4	190	21	12.8 (8.3–19.6)	64.2 (60.1–68.1)
	4–5	139	14	11.3 (6.7–19.0)	68.0 (63.8–71.9)
	5–6	109	7	7.8 (3.7–16.4)	70.3 (66.0–74.2)
	6–7	71	0	0.0	70.3 (66.0–74.2)

Estimates of yearly probability for HIV rebound determined by computing Hazard Rate via the Life Table method and cumulative probability by Kaplan–Meier (SAS 9.3, Cary, NC). Analysis restricted to the first sequence of each patient with consecutive viral loads less than the lower limit of quantitation. Sequences were right-censored 43 times because the testing interval exceeded 390 days and 317 times when the observation period ended 31 December 2011

After three years of virologic suppression, the risk of viral rebound dropped significantly. For patients who achieved three to five years of virologic suppression, the risk of failure were 12.8 % (95 % CI 7.3–18.2) overall and 9.7 % for CD4 >300 in the year following three years of virologic suppression, 11.3 % overall and 7.7 % for CD4 >300 in the year following four years of virologic suppression, 7.8 % overall and 6.5 % for CD4 >300 in the year following five years of virologic suppression. For 79 patients in both CD4 strata who achieved 5.7 years of virologic suppression, none had viral rebound at a median of 10 months of follow-up.

### Risk of Viral Rebound by Year for Analysis B, >200

The risk of viral rebound by year, defined as >200 viral copies/ml (Table 3 and Fig. 2), in the first three years was high. When stratified by CD4 cell count, patients with >300 cells/µl were at lower risk of viral rebound than those with <300 cells/µl only in the first year following virologic suppression. Thereafter, differences by CD4 strata did not achieve statistical significance. The risk of viral rebound dropped significantly in the year following three years of virologic suppression and thereafter. For 119 patients in both CD4 strata who achieved 5.7 years of virologic suppression, none had viral rebound at a median of 10 months of follow-up.

**Table 3:** Probability of HIV rebound above 200 copies/ml during continuous suppression

Initial CD4 count	Years of HIV suppression (year)	No. at start of year	No. of rebounds	Yearly % probability (95 % CI)	Cumulative % probability (95 % CI)
<300 cells/μl	0–1	248	79	40.8 (32.9–50.7)	34.6 (28.7–41.0)
	1–2	139	28	25.0 (17.3–36.1)	48.5 (41.8–55.2)
	2–3	85	16	23.2 (14.3–37.7)	59.3 (52.0–62.0)
	3–4	53	4	8.8 (3.3–23.4)	62.9 (55.3–69.8)
	4–5	38	3	9.2 (3.0–28.6)	66.1 (58.2–73.2)
	5–6	27	3	13.3 (4.3–41.2)	70.2 (61.7–77.5)
	6–7	18	0	0.0	70.2 (61.7–77.5)
>300 cells/μl	0–1	543	92	20.0 (19.2–24.5)	18.2 (15.0–21.8)
	1–2	377	54	16.8 (16.1–21.9)	30.8 (26.7–35.2)
	2–3	266	30	12.8 (12.2–18.3)	39.2 (34.6–43.9)
	3–4	202	11	6.1 (5.9–11.0)	42.8 (38.0–47.7)
	4–5	159	7	4.8 (4.7–10.1)	45.4 (40.4–50.4)
	5–6	131	7	6.3 (6.0–13.3)	48.6 (43.4–53.9)
	6–7	90	0	0.0	48.6 (43.4–53.9)
All combined	0–1	791	171	26.2 (22.5–30.4)	23.3 (20.4–26.5)
	1–2	516	82	18.9 (15.2–23.5)	36.3 (32.8–40.0)
	2–3	351	46	15.2 (11.4–20.3)	45.4 (41.4–49.4)
	3–4	255	15	6.6 (4.0–11.0)	48.9 (44.8–53.0)
	4–5	197	10	5.6 (3.0–10.5)	51.6 (47.4–55.8)
	5–6	158	10	7.5 (4.0–14.0)	55.0 (50.6–59.3)
	6–7	108	0	0.0	55.0 (50.6–59.3)

Estimates of yearly probability for HIV rebound determined by computing Hazard Rate via the Life Table method and cumulative probability by Kaplan–Meier analysis (SAS 9.3, Cary, NC). Analysis restricted to the first sequence of each patient with consecutive viral loads less than the lower limit of quantitation. Sequences were right-censored 53 times because the testing interval exceeded 390 days and 404 times when the observation period ended 31 December 2011

### Comparison of Viral Rebound Defined as >LLOQ to Viral Rebound Defined as >200 copies/ml

For both CD4 strata, the yearly risk of rebound in years one to three was approximately 10 % higher for the more stringent definition of >LLOQ. However, following three years of virologic suppression for the CD4 >300 strata, the yearly risk of rebound was equivalent regardless of definition of viral rebound.

## Discussion

The primary risk of inadequate viral load monitoring is undetected viral rebound with potential immunologic decline, immune activation and progressive selection of resistance mutations that limit antiretroviral options. Although we are informed by data from clinical trials, we conducted this study to better understand the risk of viral rebound relative to time with virologic suppression in a complex outpatient clinic environment. Our findings have relevance for HIV clinic practices and further inform recommendations for the appropriate frequency of viral load monitoring and further consideration in appropriate timing for intensive adherence strategies.

Our prior examinations of CD4 cell count and viral loads from 1999 to 2011 demonstrated considerable improvement in median CD4 cell count and the percentage of patients with virologic suppression [2] as also demonstrated elsewhere [3]. Now, in the era of potent antiretroviral therapy and capacity for genotypic resistance testing to guide therapy, the occurrence of viral rebound may reflect our challenges with retention in care and adherence to antiretrovirals [4]. It is therefore particularly disappointing that the risk of viral rebound is high out to three years. The clinic from which this data is derived provides both HIV care and primary care, has a “medical home” approach with a nurse practitioner– physician team for each patient, social workers and a clinical pharmacist on site as well as availability of an HIV psychologist. Though this model improves outcomes in the engagement in care continuum [5, 6], we, like others have demonstrated this high early risk for viral rebound, [4, 7–11] indicating that further refinement of approach is warranted. These findings support not only the suggested higher frequency of early viral load monitoring, but also highlights the period of time when additional strategies are needed to keep patients in care and on treatment.

When stratified by CD4 cell count, patients with CD4 <300 had a nearly double risk of viral rebound. Higher rates of viral rebound among patients with a low CD4 cell count in the first three years following virologic suppression is not unexpected. Patients with a low CD4 cell count (<300 cells/µl) may represent a population who may have late HIV diagnosis, with very low nadir CD4 and immune restoration failure due to inability to reconstitute depleted T cell populations despite virologic suppression. Some also have a low CD4 due to a co-morbidity such as Hepatitis C and cirrhosis despite virologic suppression. However, those with a CD4 cell count <300 are over-represented by those who are under-treated for HIV due to the failure to engage in care and attend visits to the clinic (even with two viral load measurements in 390 days, engagement in care cannot be assumed); and those who come to their visits but fail to take prescribed antiretroviral therapy.

On the other hand, after 3 years of sustained virologic suppression, the risk of rebound is quite low and our confidence in a twice yearly monitoring strategy improves. This risk declines even further by 6 years, an observation also seen by Lima et al. [4]. In our analysis, viral rebound was not seen after 5.7 years of virologic suppression among 119 patients at risk for a median of 10 months. For those patients with demonstrated consistent adherence and engagement in care for five to 6 years, even further reduction in monitoring may be rational [12].

We examined the risk of viral rebound with rebound defined both as >LLOQ and >200 copies/ml. Although the >200 definition is intended to allow for clinically insignificant “viral blips” and follows the antiretroviral guideline definition [1], recent literature suggests an increased risk of early viral rebound even with very low replication of HIV [13, 14]. A more stringent definition of viral rebound was therefore also examined. Although we did not compare the risk of viral rebound at “not detected” compared to <LLOQ, our data demonstrated that once viral suppression was achieved for five to 6 years, annual failure risk was similar regardless of rebound definition and CD4 stratification. Benzie and Lima have demonstrated durability regardless of adherence or previous treatment failures once around 6 years of suppression are achieved [14–16], thus true durability at the HIV population level may be best defined after five to six years of virologic suppression.

For the individual patient, clinician decisions as to frequency of viral load monitoring should be informed by psychosocial and neurobehavioral factors [1, 17] and self-reported adherence, pharmacy refill data or adherence monitoring [18–20]. But the findings of this analysis remind us to have caution; we should not assume durable virologic suppression after one year or even two years of virologic suppression, but carefully assess the likelihood of viral rebound. Yet, six or more years of undetectable viral loads for the “right” patient might even allow an annual viral load monitoring strategy.

Viral load monitoring is costly and particularly prohibitive in resource-limited countries. We previously demonstrated that frequent CD4 monitoring among patients with CD4 ≥300 cells/µl and virologic suppression was not necessary [21]. A less intensive strategy for viral load monitoring after truly durable virologic suppression has further significant economic implications in both resource rich and resource poor nations and warrants further prospective evaluation.

Our study had several limitations. This was a retrospective evaluation from a single, urban medical center caring for predominantly African-American men. The risk of viral rebound by antiretroviral regimen was not examined. This analysis intended to address risk for the population overall, for all patients who achieved initial virologic suppression. We examined data beginning in 2005 when efavirenz and simpler once daily regimens were more widely in use to reflect the “current” era of ART. Although the higher barrier to mutation of the newer once daily integrase strand transfer inhibitors may reduce the risk of viral mutations associated with nonadherence compared to once daily NNRTI regimens, the findings here still provide relevant guidance to a rational approach to viral load monitoring and to timing of strategies to improve adherence and reduce the risk of viral rebound.

## Conclusions

In conclusion, HIV-infected patients in our urban clinic had high rates of viral rebound in the first three years following virologic suppression, highlighting the time when targeted efforts to assure antiretroviral adherence may be particularly meaningful. On the other hand, this data demonstrated that once virologic suppression is achieved and sustained for three years, the risk for rebound declines substantially, supporting guidance for reduced monitoring particularly for patients with CD4 cell counts >300 cells/μL. As we enter eighteen years post approval of potent antiretroviral regimens, better defining the relative risks for viral rebound allows better and more focused use of resources and improvement of our capacity to achieve truly durable virologic suppression in all patients initiating antiretroviral therapy.
